# The role of losses in determining hyperbolic material figures of merit

**DOI:** 10.1038/s41598-024-74398-1

**Published:** 2024-10-24

**Authors:** E. M. Jackson, J. G. Tischler, D. C. Ratchford, C. T. Ellis

**Affiliations:** 1 Naval Research Laboratory, Washington, DC 20375 USA; 2 Physics and Astronomy Department, University of Oklahoma, Norman, Oklahoma 73019 USA; 3 Center for Quantum Research and Technology, University of Oklahoma, Norman, Oklahoma 73019 USA

**Keywords:** Optical physics, Optical materials and structures, Metamaterials, Nanophotonics and plasmonics

## Abstract

Uniaxial materials have achieved new prominence in photonics because they can have hyperbolic spectral regions with metallic (ε<0) and dielectric (ε>0) permittivities along different crystal axes. In the lossless case, this results in an open hyperboloid dispersion relation, allowing materials to support highly confined modes with extremely large wavevectors. However, even small losses change the character of the hyperbolic dispersion from open hyperboloids to closed surfaces with finite maximum *k, significantly limiting the extent to which highly-confined modes can be achieved.* Here, we derive a simple analytic formula for the dispersion relation in the presence of loss and show that for some typical materials the maximum wavevector in hyperbolic materials is roughly ten times the free-space. The scaling of the maximum wavevector is derived, and it is shown that there is a universal scaling relation between the propagation length and the wavelength, which implies that the shortest wavelengths in any hyperbolic material are strongly attenuated.

**Supplementary Information:**

The online version contains supplementary material available at 10.1038/s41598-024-74398-1.

## Introduction

There has been a great interest in hyperbolic materials and metamaterials with metallic permittivity (ε<0) along one axis and dielectric permittivity along another (ε>0), resulting in a hyperboloid dispersion relation. Various applications have been proposed that rely on the large wavevectors (*k*) that result from this unique dispersion, including hyperlensing^1,2^, optical circuitry^3^, and nanolithography^4^, which take advantage of the high-k waves to image small features. The hyperboloid dispersion also results in a photonic density of states proportional to k_m_^3^, which can be much larger than in materials that do not exhibit hyperbolicity^5,6^, enabling applications such as thermal emission engineering^7–9^, spontaneous emission engineering^10,11^, and single-photon sources^12^. However, the promise that hyperbolic materials offer these applications is limited by losses. To realistically estimate the potential impacts that hyperbolic materials offer these technologies, it is necessary to understand how losses affect the dispersion. Here, we derive a simple analytic expression for the dispersion relation in the presence of loss for materials with a uniaxial, complex permittivity or permeability. This applies to most metamaterial-based^13,14^ and natural hyperbolic materials^15–18^. Overall, we show that the presence of loss results in a closed dispersion relation with a maximum wavevector, k_m_, that scales with the loss. This limit on k is independent of the limit due to the atomic or superlattice periodicity of the hyperbolic material, and we show that in many cases it is the loss that constrains k_m_^3^.

These results have important implications for the performance of hyperbolic materials in a variety of applications. Hyperbolic materials are often touted for their ability to achieve subwavelength control of light due to the large k values allowed. For example, the size of optical cavities can be reduced to ~1/k_m_, much smaller than the free space wavelength^19^. Our results clearly show that the loss of the material is often the most stringent constraint on k_m_ and therefore the primary limiting factor on the achievable compression. Furthermore, we show that within a hyperbolic material at constant frequency, the λ and the propagation length, L_p_, are correlated with a functional dependence characterized by a parameter Δ. The shortest wavelengths are the most heavily attenuated. This impacts the resonant quality factor which is proportional to L_p_^20^. The optimal cavity design must take both compression and quality factor into account.

As such, Δ and k_m_ are important figures of merit for hyperbolic materials. The simple analytic formulas derived here for these figures of merit will enable rapid evaluation of hyperbolic materials and aid in the identification of the best design parameters for a given material and application.

For simplicity, we restrict ourselves to uniaxial materials where spatially uniform permittivity and permeability tensors can be defined. The permeability and permittivity are both assumed to be diagonal with the extraordinary axis along the z-axis. In this case, the material has cylindrical symmetry, and the permittivity has components ε_x_= ε_y_≡ ε_r_ and ε_z_. Similarly, the permeability components are μ_r_ and μ_z_. Then, for a plane wave, Maxwell’s equations yield the dispersion relation^21^:

1a $${\text{TM mode:}}\quad k_{0}^{2} = \frac{{k_{r}^{2} }}{{\mu_{{\text{r}}} \varepsilon_{z} }} + \frac{{k_{z}^{2} }}{{\mu_{{\text{r}}} \varepsilon_{r} }}$$

1b $${\text{TE mode:}}\quad k_{0}^{2} = \frac{{k_{r}^{2} }}{{\mu_{z} \varepsilon_{r} }} + \frac{{k_{z}^{2} }}{{\mu_{{\text{r}}} \varepsilon_{r} }}$$

where k_0_=ω/c is the free space wavevector and k_r_ and k_z_ are the wavevectors in the x-y plane (ordinary axes) and along the z (extraordinary axis), respectively. The full solution is then found by rotating the solution around the z axis. The electric field of the TM mode is in the plane of incidence defined by k and z, and the B field is perpendicular to it. Similarly, for the TE mode, the B field is in the plane of incidence and the E field is perpendicular to it. The dispersion relations of both modes are described by the same functional form with the roles of ε and μ reversed. We therefore proceed by solving for the dispersion of the TM mode. The dispersion of the TE mode can be obtained by simple substitution μ_j_ for ε_i_ and vice versa, where the index j=r,z. We denote the real and imaginary parts of ε and μ by subscripts 1 and 2, respectively, i.e. Re(ε_j_)≡ ε_j1_ and Im(ε_j_)≡ ε_j2_.

**Fig. 1:**
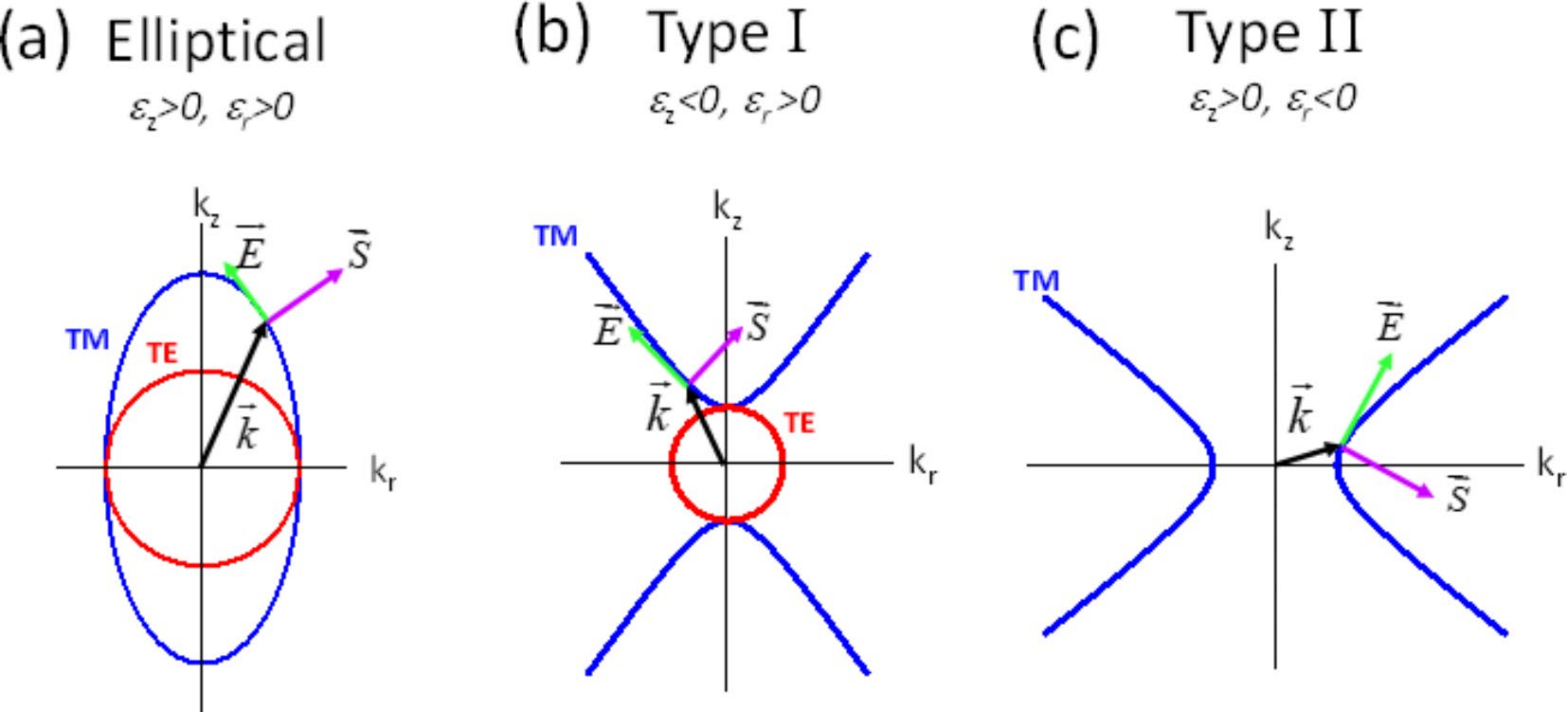
2D slice of the lossless dispersion curves for a nonmagnetic material, exhibiting a) Elliptical b) Type I Hyperbolic c) Type II Hyperbolic behavior. The full dispersion is obtained by rotating around the k_z_ axis. Only the E-field of the TM mode is shown. The TE mode E-field and TM mode B fields are out of the page. The dispersion of both modes is purely real.

When the real parts of ε are both negative the solution is trivial and there is no propagation. However, there are three nontrivial cases of interest: elliptical (ε_r1_ > 0, ε_z1_ > 0), Type I hyperbolic (ε_r1_ > 0, ε_z1_ < 0), and Type II hyperbolic (ε_r1_< 0, ε_z1_ > 0). Representative illustrations of the lossless dispersion relations for each case are shown in Fig. 1. In all cases, the electric field is tangent to the dispersion curve, and the Poynting vector is along the outward normal to it.

Although Eq. 1 is general for all uniaxial materials, Fig. 1 is correct only if the permittivities are purely real. However, since all materials have losses, the permittivities are always complex. Then k must also be complex such that the real part of the wavevector Re(k) is 2π/λ and the imaginary part Im(k) is the inverse of L_p_, i.e., the distance over which the wave’s magnitude decreases by 1/e. We begin our analysis by solving Eq. 1 to determine Re(k) and Im(k) for hyperbolic materials.

We write the complex permittivities and permeabilities as $|\varepsilon_{j} |e^{{ - i\gamma_{\varepsilon j} }}$ and $|\mu_{j} |e^{{ - i\gamma_{\mu j} }}$, respectively, where j=z,r. Here, the signs of the permittivities are reflected in the corresponding argument γ. In the elliptical case, all γs are in the first quadrant (i.e., 0<γ_εj,_ γ_μj_<π/2). In the hyperbolic cases, we assume both γ_μj_ and the γ of the dielectric permittivity will be in the first quadrant and the γ of the metal permittivity will be in the 2^nd^ quadrant (e.g. Type I: 0<γ_εr,_ γ_μj_ <π/2 and π/2<γ_εz_<π & Type II: 0<γ_εz,_ γ_μj_ <π/2 and π/2<γ_εr_<π). As the losses approach zero, γ for the dielectric and metallic axes approach 0 and π, respectively.

**Fig. 2:**
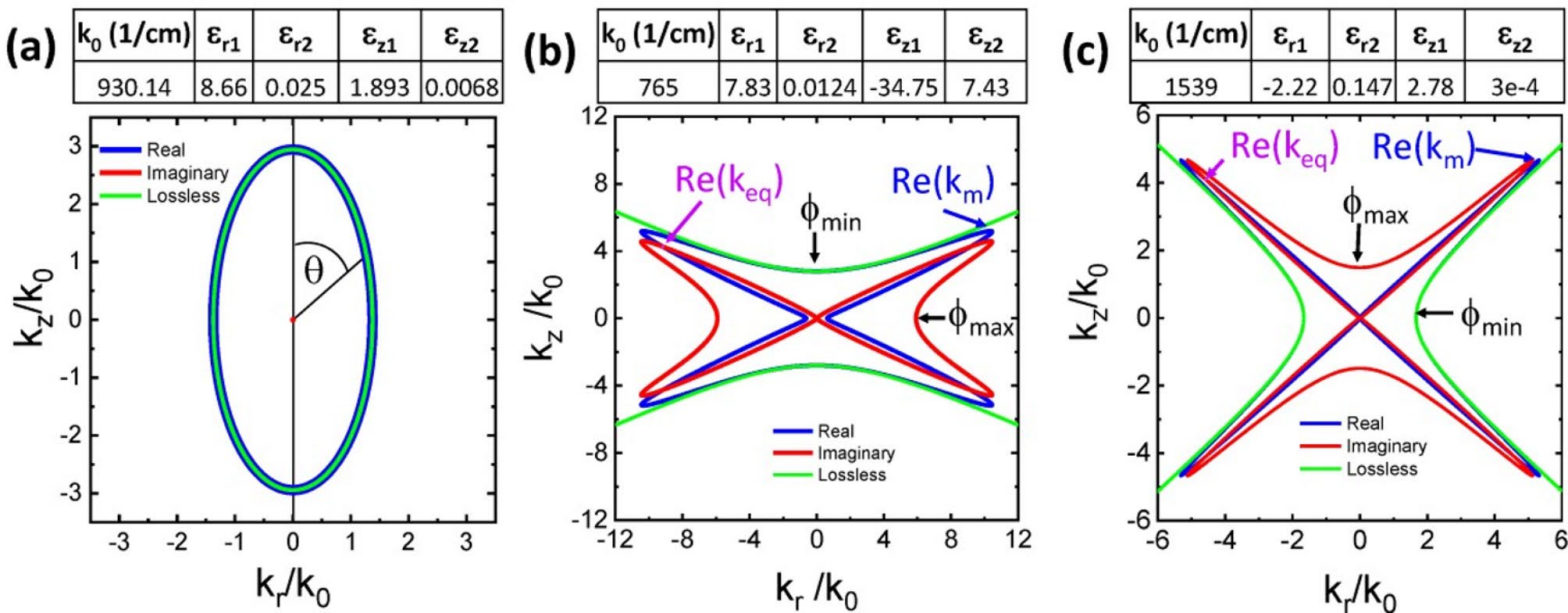
Uniaxial Dispersion of hBN: the full 3d dispersion is obtained by rotating around the z axis (a) Elliptical regime. The propagation angle θ is defined as shown. (b) Type I hyperbolic regime. The minimum and maximum phases are at 0° and 90°, repectively. (c) Type II hyperbolic regime. The minimum and maximum phases are at 90° and 0°, respectively. In the lossy cases, angles where k_imag_(θ)(red line)>k_real_(θ) (blue line) correspond to the nonpropagating angles of the lossless curve (see also Fig. 4a).

Similarly, we rewrite the wavevector components as $k_{j} = |k_{j} |e^{{i\phi_{j} }}$, where ϕ_j_ is the phase for the two axes. The real and imaginary parts of k must be collinear, i.e. |k_z_|sin(γ_εz_)/|k_r_|sin(γ_εr_)=|k_z_|cos(γ_εz_)/|k_r_|cos(γ_εr_). If not, the fields will grow exponentially in the direction perpendicular to the propagation, resulting in a divergence. This condition is satisfied when $\upphi _{{\text{z}}} =\upphi _{{\text{r}}} \equiv\upphi$ so $\vec{k} = |\vec{k}|e^{i\phi } \hat{k}$ where $\hat{k}$ is a unit vector in the direction of $\vec{k}$. With these definitions, we find the following solution for Eq. 1 (see “Supplementary Material” for details):

2a $$\left( {\begin{array}{*{20}c} {{{k_{r} } \mathord{\left/ {\vphantom {{k_{r} } {k_{0} }}} \right. \kern-0pt} {k_{0} }}} \\ {{{k_{z} } \mathord{\left/ {\vphantom {{k_{z} } {k_{0} }}} \right. \kern-0pt} {k_{0} }}} \\ \end{array} } \right) = e^{i\phi } f(\theta )\left( {\begin{array}{*{20}c} {\sin (\theta )} \\ {\cos (\theta )} \\ \end{array} } \right)$$

2b $$\tan (2\phi ) = \frac{{\left| {\varepsilon_{r} } \right|\sin (\gamma_{\varepsilon z} + \gamma_{\mu r} )\sin^{2} (\theta ) + \left| {\varepsilon_{z} } \right|\sin (\gamma_{\varepsilon r} + \gamma_{\mu r} )\cos^{2} (\theta )}}{{\left| {\varepsilon_{r} } \right|\cos (\gamma_{\varepsilon z} + \gamma_{\mu r} )\sin^{2} (\theta ) + \left| {\varepsilon_{z} } \right|\cos (\gamma_{\varepsilon r} + \gamma_{\mu r} )\cos^{2} (\theta )}}$$

2c $$f(\theta ) = \sqrt {\left| {\mu_{r} } \right|} \left( {\frac{{\sin^{4} (\theta )}}{{\left| {\varepsilon_{z} } \right|^{2} }} + \frac{{\cos^{4} (\theta )}}{{\left| {\varepsilon_{r} } \right|^{2} }} + 2\cos (\gamma_{\varepsilon z} - \gamma_{\varepsilon x} )\frac{{\cos^{2} (\theta )\sin^{2} (\theta )}}{{\left| {\varepsilon_{z} } \right|\left| {\varepsilon_{r} } \right|}}} \right)^{ - 1/4}$$

where θ =atan(k_r_/k_z_) is the polar angle of the wavevector (see Fig. 2a for graphical representation).

We illustrate the properties of Eq. 2 for the nonmagnetic (μ_r_=1 and γ_μr_=0) material hexagonal boron nitride (hBN), which exhibits simultaneous metallic and dielectric behavior along different crystal axes within the reststrahlen bands that are bounded by the transverse optical (TO) and longitudinal optical (LO) phonon frequencies. Representative dispersion curves at frequencies exhibiting the three non-trivial behaviors (elliptical, Type I hyperbolic, and Type II hyperbolic) are shown in Fig. 2a-c, using the permittivities given in Table 1. Figure 2a shows that in the elliptical band, Eq. 2 reproduces the standard lossless result if the loss terms are small. Significant deviation from the lossless case occurs only near the TO and LO phonon frequencies of the lower and upper reststrahlen bands, where the imaginary part of the permittivity (ε_z2_ or ε_r2_) becomes larger than the corresponding real part (ε_z1_ or ε_r1_).

**Table 1:** Permittivities and dispersion curve properties.

Material	Type	k_0_	ε_r1_	ε_r2_	ε_z1_	ε_z2_	Δ	θ_km_	Re(k_km_/k_0_)
		(1/cm)						(deg)	
Alumina/Au Layers	Type II	13986	-8.2	0.9	12.5	0.5	-0.05	52.12	9.64^a^
Alumina/Au rods	Type II	18416	-4.0	0.71	1.29	0.04	-0.019	30.74	4.12^b^
Alumina/Ag rods	Type I	11111	5.0	0.03	-7.5	0.6	-0.028	50.10	9.82^c^
Alumina/Ag rods	Type I	8333	4.9	0.017	-16	1.3	-0.016	60.54	12.75^c^
Alumina/Ag rods	Type I	6667	4.8	0.012	-25	3.0	-0.017	65.75	12.71^c^
HfN/ScN layers	Type I	19568	1.2	2.35	-5.4	12	1.32	70.7	2.14^d^
HfN/ScN layers	Type II	8322	-17.4	6.35	8	0.4	-0.07	35.8	6.73^d^
TiN/ScN layers	Type I	14298	0.865	1.03	-6.17	24.0	1.09	82.3	3.16^e^
TiN/ScN layers	Type II	6661	-17.6	6.86	15.9	0.875	-0.14	45.6	7.59^e^
TiN/ScN layers	Type II	11111	-2.8	1.5	4	0	-0.28	51.8	3.32^f^
Calcite	Type I	871	3.25	0.012	-21.57	68.35	-0.039	78.64	7.11
Calcite	Type I	872	3.26	0.012	-23.81	9.94	-0.04	69.04	7.02
Calcite	Type I	879	3.27	0.012	-2.226	0.23	-0.06	38.77	5.94
Calcite	Type I	885	3.29	0.012	-0.459	0.076	-0.14	19.69	3.98
Calcite	Type I	888	3.30	0.012	-0.036	0.052	-1.40	5.04	1.95
Calcite	Elliptical	888.5	3.30	0.0124	0.020	0.049	2.52	0	1.82
Calcite	Elliptical	1100	3.97	0.023	1.88	1.5E-3	-0.0037	0	1.99
Calcite	Elliptical	1402	10.23	87.69	2.02	6.1E-4	-0.022	5.96	7.64
Calcite	Type II	1405	-35.24	66.80	2.02	6.0E-4	-0.023	9.26	7.54
Calcite	Type II	1410	-36.37	23.57	2.02	6.0E-4	-0.024	13.12	7.38
Calcite	Type II	1450	-5.73	0.813	2.03	5.5E-4	-0.036	31.55	6.06
Calcite	Type II	1500	-1.43	0.195	2.04	4.9E-4	-0.079	50.97	4.18
Calcite	Type II	1550	-0.027	0.086	2.04	4.4E-4	-2.78	84.28	1.46
hBN	Elliptical	930	8.66	0.025	1.89	0.068	0.004	0	2.94
hBN	Type I	765	7.83	0.0124	-34.7	7.43	-0.037	63.7	11.7
hBN	Type II	1539	-2.22	0.147	2.78	3.0E-4	-0.037	48.7	7.1

^a^Reference^5^, ^b^Reference^19^, ^c^Reference^20^, ^d^Reference^21^, Reference^22^, ^f^Reference^23^.

In contrast to the elliptical case, even small losses have a significant impact on the hyperbolic dispersion, where the unbounded, open hyperbolic dispersion that occurs in the lossless case becomes closed with finite maximum *k* in the presence of losses. Figure 2b and c show representative cases for the Type I and II hyperbolic bands of hBN, respectively. For both types, the error in assuming lossless dispersion increases as the wavevector angle θ moves away from the center of the hyperbola. If the loss is small, the real part of the dispersion is approximately hyperbolic until Re(k) approaches its maximum (k_m_). However, as shown below, the absorption (Im(k)) also increases very rapidly near this point resulting in short propagation lengths for the shortest wavelengths (largest wavevectors). In the case where losses are large, the deviation between the lossy and lossless dispersion curves occurs well before approaching k_m_ (see Supplemental Figs. S1 and S2 for examples). As such, when evaluating hyperbolic systems, it is important to carefully consider the material losses and the range over which the system can be well-approximated by the lossless model. Furthermore, it is important to understand that losses limit the maximum theoretically achievable wavevector since the presence of losses transforms the unbounded dispersion of lossless systems to a bounded one.

Although it seems natural to determine the maximum of Re(k) by taking a derivative of Eq. 2 with respect to θ, this leads to equations that are extremely difficult to solve analytically. We overcome this challenge by rewriting the dispersion (Eq. 2) as a parametric function of the phase ϕ, which allows the derivative with respect to ϕ to be solved analytically. The parametric form of Eq. 2 is given by (see “Supplementary Material” for details):

3a $$\frac{k}{{k_{0} }} = \sqrt {\left| {\mu_{r} } \right|} e^{i\phi } \sqrt {\frac{{\left( {\left| {\varepsilon_{r} } \right|\sin (\gamma_{\varepsilon z} + \gamma_{\mu r} ) - \left| {\varepsilon_{z} } \right|\sin (\gamma_{\varepsilon r} + \gamma_{\mu r} )} \right)\cos (2\phi ) - \left( {\left| {\varepsilon_{r} } \right|\cos (\gamma_{\varepsilon z} + \gamma_{\mu r} ) - \left| {\varepsilon_{z} } \right|\cos (\gamma_{\varepsilon r} + \gamma_{\mu r} )} \right)\sin (2\phi )}}{{\sin (\gamma_{\varepsilon z} - \gamma_{\varepsilon r} )}}}$$

3b $$\tan (\theta ) = \sqrt {\left( {\frac{{\left| {\varepsilon_{z} } \right|}}{{\left| {\varepsilon_{r} } \right|}}} \right)\frac{{\sin (\gamma_{\varepsilon r} + \gamma_{\mu r} ) - \tan (2\phi )\cos (\gamma_{\varepsilon r} + \gamma_{\mu r} )}}{{\tan (2\phi )\cos (\gamma_{\varepsilon z} + \gamma_{\mu r} ) - \sin (\gamma_{\varepsilon z} + \gamma_{\mu r} )}}}$$

where sin(γ_εz_ − γ_εr_) ≠ 0. Equation 3 is equivalent to Eq. 2 if the limits of ϕ are chosen appropriately.

From Eq. 2b, we find that the phase ϕ equals (γ_εr_+γ_μr_)/2 at θ=0° and (γ_εz_+γ_μr_)/2 at θ =90°. It increases (Type I) or decreases (Type II) monotonically with θ between these 2 points. Therefore Eq. 3 reproduces the 1^st^ quadrant of the dispersion curve for Type I materials with ϕ_min_≡(γ_εr_+γ_μr_)/2≤ϕ ≤(γ_εz_+γ_μr_)/2≡ϕ_max_. ϕ_min_ and ϕ_max_ are reversed for Type 2 materials, i.e. ϕ_min_≡(γ_εz_+γ_μr_)/2 and ϕ_max_ ≡(γ_εr_+γ_μr_)/2. The remainder of the dispersion curve is determined by symmetry.

We define the magnitude of k where the real part of k is maximal to be k_m_ (see Fig. 2b,c) and determine the phase ϕ_km_ where it occurs by setting ∂Re(k)/∂ϕ=0. The solution is (see “Supplementary Material”):

4a $$\phi_{{{\text{km}}}} = \frac{\pi }{6} + \frac{1}{3}\tan^{ - 1} \left( \Delta \right)$$

where

4b $$\Delta = \frac{{\left| {\varepsilon_{r} } \right|\sin (\gamma_{\varepsilon z} + \gamma_{\mu r} ) - \left| {\varepsilon_{z} } \right|\sin (\gamma_{\varepsilon r} + \gamma_{\mu r} )}}{{\left| {\varepsilon_{r} } \right|\cos (\gamma_{\varepsilon z} + \gamma_{\mu r} ) - \left| {\varepsilon_{z} } \right|\cos (\gamma_{\varepsilon r} + \gamma_{\mu r} )}}$$

In the elliptical case, this solution is not typically allowed because ϕ_km_ is larger than the maximum allowed phase. In this case, Re(k) has no maximum between θ =0° and 90°. Instead, its maximum is at either the θ =0° or the θ =90° endpoint. However, in hyperbolic materials the phase typically ranges from close to 0 to almost π/2 so ϕ_km_ is within the allowed range of ϕ and Re(k) has a maximum between θ =0° and 90°.

**Fig. 3:**
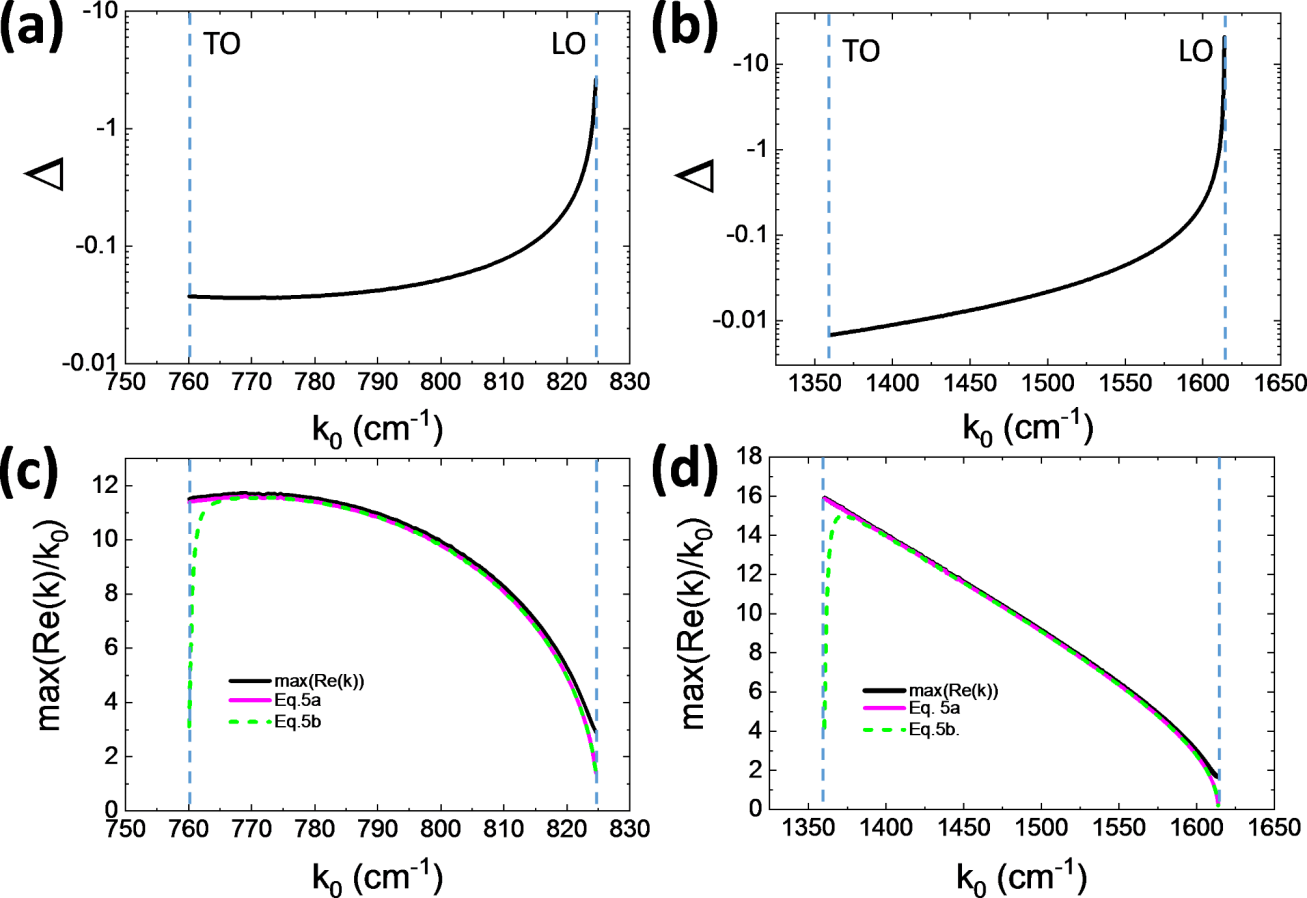
Δ and the maximum of the real part of k. The LO and TO frequencies are marked by dashed lines. (a) Δ for Type I hBN and (b) Type II hBN. (c) Re(k) at the angle where it is maximum for hBN in its (c) Type I band and (d) Type II spectral bands. The black lines are from the full solution. The magenta and green lines show Eqs.5a and 5b, respectively. The small angle approximation for γ_m_ fails at both band edges as the real part of the metallic (Re(ε_m_)<0) permittivity goes to zero.

In hyperbolic materials, Δ is generally small. The cosine terms have opposite signs, and in low loss materials have magnitudes close to 1, so the denominator is approximately the sum of the permittivity magnitudes. The sine terms are small and are both positive, so the numerator is a difference of 2 small terms. This is seen in Fig. 3a,b which show Δ for Type I and Type II hBN, respectively. In both cases, Δ is small over most of the range. Only near the high frequency band edges where the metallic (Re(ε_m_)<0) permittivity |ε_m_|→0 and ε_m2_/ε_m1_ simultaneously becomes large does Δ become large. However, under these conditions Re(k_m_) is relatively small. Over the more interesting majority of the frequency range we can take ϕ_km_ ≈ π/6.

Substituting ϕ_km_ ≈ π/6 into Eq. 3a yields the maximum wavevector:

5a $$\frac{{{\text{Re}} (k_{m} )}}{{k_{0} }} = \frac{{\lambda_{0} }}{{\lambda_{\min } }} \approx \frac{{3^{3/4} \sqrt {\left| {\mu_{r} } \right|} }}{2\sqrt 2 }\sqrt {\frac{{\left( {\left| {\varepsilon_{z} } \right|\cos (\gamma_{\varepsilon r} + \gamma_{\mu r} ) - \left| {\varepsilon_{r} } \right|\cos (\gamma_{\varepsilon z} + \gamma_{\mu r} )} \right)}}{{\sin (\gamma_{\varepsilon z} - \gamma_{\varepsilon r} )}}}$$

5b $$\frac{{{\text{Re}} (k_{m} )}}{{k_{0} }} \approx \frac{{3^{3/4} \sqrt {\left| {\mu_{r} } \right|} }}{2\sqrt 2 }\sqrt {\frac{{\left| {\varepsilon_{z} } \right| + \left| {\varepsilon_{r} } \right|}}{{\left| {\varepsilon_{z2} /\varepsilon_{z1} } \right| + \left| {\varepsilon_{r2} /\varepsilon_{r1} } \right|}}}$$

where Eq. 5b follows a 1^st^ order expansion of Eq. 5a (see “Supplementary Material” for details). Therefore, the minimum wavelength achievable by a nonmagnetic, hyperbolic material scales as λ_min_∝λ_0_(|ε_r2_/ε_r1_|+|ε_z2_/ε_z1_|)^1/2^/(|ε_r_|+|ε_z_|)^1/2^ where λ_0_ is the free space wavelength. From this, we see that there are 2 ways to achieve small λ_min_. If the permittivity |ε_r_|+|ε_z_| is held constant, the loss term (|ε_r2_/ε_r1_|+|ε_z2_/ε_z1_|) must be decreased. In this case, due to the square root, a reduction in λ_min_ by a factor of 10 requires reducing the losses by a factor 100. Alternatively, either the metallic or dielectric permittivity can be increased.

Polar dielectric crystals are among the lowest loss hyperbolic materials ^16^. As an example of the wavelength compression that can be achieved in practice, in Fig. 3c,d, we plot the maximum of Re(k) of hBN as a function of frequency for the two hyperbolic regimes and compare the result to the small Δ and small angle approximations. Near the high frequency band edges, both approximations break down because Δ becomes large as the real part of the metallic permittivity ε_m1_→0 and the imaginary part ε_m2_ becomes small more slowly. Near the low frequency band edges ε_m1_→0 but ε_m2_ grows rapidly. The small angle approximation fails, but Δ remains small because |ε_m_|>>1. The maximum wavelength compression is less than a factor of 20 times smaller than the free space wavevector. The maximum compression for several typical metamaterial examples from the literature are shown in Table 1^5,22–26^. In each case analyzed, the maximum compression is ~10. It is often discussed in the literature that for metamaterials the maximum achievable wavevector is limited by the lattice period as it becomes comparable to the wavelength.^14,17^ However, losses represent another limit on the maximum wavevector. In practice, while for some metamaterials in the visible the minimum wavelength may be limited by the superlattice period, in most cases, the primary limit on the maximum achievable compression will be due to the loss.

In addition to limiting the maximum value of k, losses also limit the propagation length of the waves. Perhaps surprisingly, we find that there is a scaling relation between L_p_ and λ whose shape depends on the permittivity only through Δ and a λ_eq_ defined below.

We define k_eq_ and λ_eq_ as the wavevector and wavelength, respectively, where the real and imaginary parts of k are equal. At this point the phase is π/4 by definition. We note that π/4 is always within the allowed range of the phase for hyperbolic materials and never within the range for elliptical materials. Taking the limit ϕ→π/4 in Eq. 3b we find that this point also corresponds to the angle θ_eq_ given by $tan{\theta }_{eq}=\left(-\left|{\varepsilon }_{z}\right|cos{\gamma }_{r}/\left|{\varepsilon }_{r}\right|cos{\gamma }_{z}\right)$. In the limit of zero losses θ_eq_ approaches the asymptotic angle of the lossless hyperbola. Substituting π/4 into Eq. 3a we find:

6 $$\frac{{\lambda_{0} }}{{\lambda_{eq} }} = \frac{{{\text{Re}} (k_{eq} )}}{{k_{0} }} = \frac{{{\text{Im}} (k_{eq} )}}{{k_{0} }} = \frac{{\sqrt {\left| {\mu_{r} } \right|} }}{\sqrt 2 }\sqrt {\frac{{\left( {\left| {\varepsilon_{z} } \right|\cos (\gamma_{\varepsilon r} + \gamma_{\mu r} ) - \left| {\varepsilon_{r} } \right|\cos (\gamma_{\varepsilon z} + \gamma_{\mu r} )} \right)}}{{\sin (\gamma_{\varepsilon z} - \gamma_{\varepsilon r} )}}}$$

Dividing Eq. 3 by Eq. 6 and recalling that Re(k)/Im(k)=cot(ϕ) we find (see “Supplementary Material” for details):

7a $$\frac{\lambda }{{\lambda_{eq} }} = \frac{{{\text{Re}} (k_{m} )}}{{{\text{Re}} (k)}} = \frac{1}{{\sqrt 2 \cos (\phi )\left[ {\sin (2\phi ) - \Delta \cos (2\phi )} \right]^{1/2} }}$$

7b $$\frac{{2\pi L_{p} }}{{\lambda_{eq} }} = \frac{{{\text{Re}} (k_{m} )}}{{{\text{Im}} (k)}} = \frac{1}{{\sqrt 2 \sin (\phi )\left[ {\sin (2\phi ) - \Delta \cos (2\phi )} \right]^{1/2} }}$$

Equation 7 describes a family of universal curves whose shapes depend only on Δ.

**Fig. 4:**
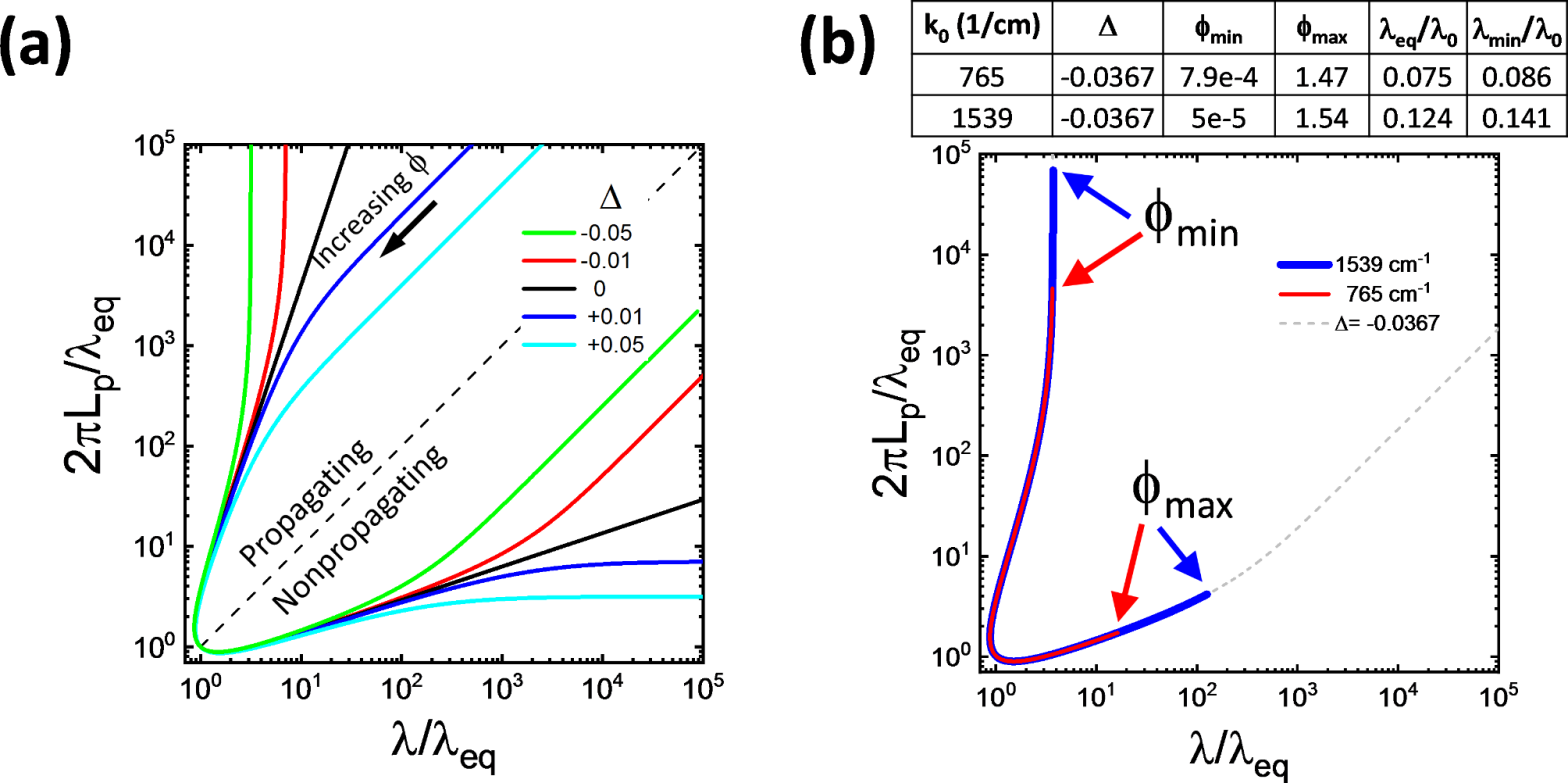
Scaling of L_p_ vs λ (a) Normalizing L_p_ and λ by λ_eq_ completely accounts for the dependence on λ_eq_. The shape of the curve depends only on Δ. λ/λ_eq_=2πL_p_/λ_eq_ is where the curves cross the dashed line. The portion of the scaling curve above the dashed line corresponds to the hyperbolic region of the dispersion and the portion below it corresponds to the lossless, nonpropagating region. The scaling curves continue to infinity, but for a given material the allowed range of ϕ is between its ϕ_min_ and ϕ_max_. b) Scaling of 2 hBN frequencies with the same Δ=-0.0367. Their scaling curves differ only in the range of ϕ despite very different λ_min_ and dispersion curves (see Fig. 2).

Figure 4a shows Eq. 7 for several values of Δ. For each Δ there are 2 branches, but the lower branch (below the dashed line in Fig. 4a) corresponds to the wavevectors that would not propagate at all in the lossless case. Even in the lossy case these wavevectors have very short propagation lengths. The scaling curves for -Δ and Δ are mirror images across the dashed line in Fig. 4a. Although in principle the curves can extend to infinity, the range of the phase that is allowed for a given material/permittivity set is limited by its specific ϕ_min_ and ϕ_max_, i.e. ϕ_min_≤ϕ≤ϕ_max_. L_p_ is maximal at the ϕ_min_ endpoint which is the phase at the center of the hyperbolic region (shown in Fig 2b,c). From there, ϕ increases monotonically, counterclockwise along the curve to the ϕ_max_ endpoint. There is a fundamental asymmetry between ϕ_min_ and ϕ_max_. ϕ_min_ is the endpoint of the propagating branch so reducing it can both increase λ_min_ and increase L_p_ for wavevectors near the center of the hyperbolic region of the dispersion. ϕ_max_ is the endpoint of the nonpropagating branch so its only effect on the upper branch, propagating wavevectors is through its effect on λ_eq_.

An infinite number of permittivity sets can produce any given Δ, and these permittivity sets can have a variety of dispersion curves. All permittivity sets with the same Δ fall on the same scaling curve regardless of the type and shape of the dispersion curve or the value of λ_eq_. An example is shown in Fig. 4b where the scaling curves for hBN at 765 and 1539 cm^-1^ are plotted. Both have Δ=-0.0367, but hBN is Type I at 765 cm^-1^ and Type II at 1539 cm^-1^. Also, λ_min_ at 765 cm^-1^ is significantly lower than at 1539 cm^-1^ due to its higher permittivity, and the dispersion at 765 cm^-1^ is much more anisotropic (see Fig. 2). Nevertheless, the scaling curves differ only in having different allowed ranges of the phase, i.e. different ϕ_min_ and ϕ_max_. Note also that at a given value of ϕ both λ/λ_eq_ and L_p_/λ_eq_ are the same for all scaling curves with the same Δ, but the position on the dispersion curve where that value of ϕ occurs may differ.

A general property of low loss hyperbolic materials is that Eq. 7 converges to the Δ=0 case when the wavelength is small (see Fig. 4a). This is because, as already argued, Δ is small in low loss materials. The Δcos(2ϕ) term inside the radical in Eq. 7a and 7b becomes important only at phases tan(2ϕ)≲|Δ| near the endpoints where ϕ→ϕ_min_ or ϕ_max_. Over most of the range of the phase sin(2ϕ)>>Δcos(2ϕ), and the Δcos(2ϕ) term can be neglected. In particular, at λ_min_ the phase is ϕ_km_≈π/6 >>Δ/2 so in the vicinity of ϕ_km_ the propagation length converges to the Δ=0 curve. As a consequence, the shortest wavelengths in any hyperbolic material are strongly attenuated. For example, when Δ=0 wavelengths shorter than 2.7λ_min_ are attenuated by 1/e after less than 10λ_min_ which for many hyperbolic materials is ~λ_0,_ as already shown.

In many materials Δ<0 (see Fig. 3a,b and Table 1 for examples) because the losses of the metallic permittivity dominate the losses of the dielectric permittivity, i.e. sin(γ_εz_)/|ε_z_|>sin(γ_εr_)/|ε_r_| in Type I materials or sin(γ_εz_)/|ε_z_|<sin(γ_εr_)/|ε_r_| in Type II materials. In this case, there is a limit on the range of propagating wavelengths (upper branch in Fig. 4a) that are possible for any material with a given Δ. The maximum propagating wavelength λ_c_ occurs at the center of the hyperbolic region where the phase is ϕ_min_. Then since ϕ_min_ > 0 we must have ${\lambda }_{c}/{\lambda }_{eq}\le 1/\sqrt{-2\Delta }$.

**Fig. 5:**
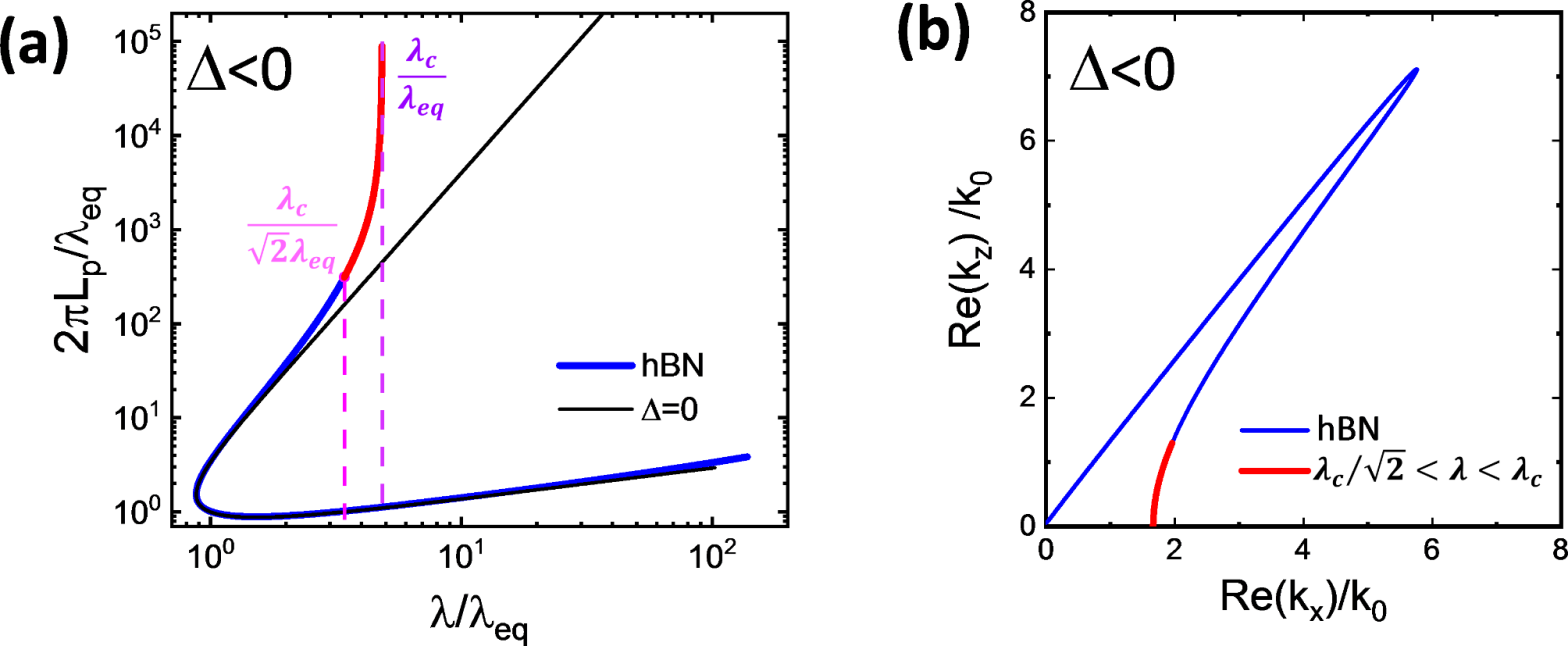
Scaling and dispersion curves of hBN at 1500 cm^-1^ (a) L_p_ vs λ. L_p_ is enhanced relative to Δ=0 between λ_c_/2^0.5^ and λ_c_ (b) Re(k) dispersion curve. The red line in (a) shows the portion of the scaling curve corresponding to the portion of the dispersion curve shown in red in (b).

Figures 4 and 5 also show that when Δ<0 wavelengths with small ϕ can have strongly enhanced L_p_ compared to Δ≥0. The wavelengths with strongly enhanced L_p_ are those λ(ϕ) where -Δcos(2ϕ)>sin(2ϕ), i.e. 0.5tan(-Δ)>ϕ≥ϕ_min_. At these small phases cos(ϕ)→1, the argument of the radical in Eq. 7a approaches |Δ|, and λ becomes almost independent of ϕ. However L_p_, becomes very large because of the 1/sin(ϕ) prefactor in Eq. 7b. If ϕ_min_<<-Δ<<1 then the argument of the radical in Eq. 7 is ≈-Δ at ϕ=ϕ_min_ and ≈-2Δ when tan(2ϕ)=-Δ. The corresponding range of wavelengths that have enhanced L_p_ is ${{\lambda_{c} } \mathord{\left/ {\vphantom {{\lambda_{c} } {\sqrt 2 }}} \right. \kern-0pt} {\sqrt 2 }} \le \lambda \le \lambda_{c}$. This is illustrated in Fig. 5a for hBN at 1500 cm^-1^ (λ_0_=6.67um). The corresponding dispersion is shown in Fig. 5b. The red portion of the dispersion with enhanced L_p_ is shown by the red line, where we see that the wavelengths with enhanced L_p_ (red portion of the scaling curve) are near the center of the hyperbolic region.

To the best of our knowledge the Δ>0 case has not been explored thus far. However, such a material might be interesting since, in principle, there is no upper limit on the wavelength and large wavelengths could be achieved even with finite losses and permittivities. The requirement to achieve a large wavelength is tan(2ϕ_min_)→Δ so that the radical in Eq. 6 approaches 0.

The lack of examples of hyperbolic materials with Δ>0 is largely because the dielectric losses in most hyperbolic materials are smaller than the metallic losses. However, another way to obtain Δ>0, which may be easier to achieve than reducing the metallic losses is to let |ε_r_|/|ε_z_|→0 (Type I) or |ε_z_|/|ε_r_|→0 (Type II). Such a material would be quite anisotropic, but the range of propagating wavelengths could be very large.

In summary, we have derived a simple analytic formula for the dispersion of lossy, uniaxial materials which also applies to uniaxial metamaterials in the effective medium approximation. In the presence of loss, the dispersion is always a closed surface, significantly limiting the maximum achievable mode compression. The maximum of the real part of the wavevector k_m_ in hyperbolic materials is found to be ~10k_0_ for several typical cases. Additionally, k_m_ scales as k_m_∝k_0_(|ε_r_|+|ε_z_|)^1/2^/(|ε_r2_/ε_r1_|+|ε_z2_/ε_z1_|)^1/2^ so increasing k_m_ by a factor of 10 requires decreasing the losses by a factor of 100. k_m_ can also be increased by increasing either of the dielectric constants. We have also shown that there is a universal scaling relation between L_p_ and λ which implies that the shortest wavelengths in any hyperbolic material are strongly attenuated. We show that these properties are characterized by parameters Δ and k_m_ which are figures of merit enabling quantitative evaluation of the performance limits of hyperbolic materials and metamaterials due to optical losses. The simple analytic formulas derived here for these figures of merit will enable rapid evaluation of hyperbolic materials and aid in the identification of the best design parameters for their application.

## Supplementary Information

Below is the link to the electronic supplementary material.

Supplementary Material 1

## Data Availability

The datasets analysed during the current study are available from the corresponding author on reasonable request.
