# Supplementary material for: The role of losses in determining hyperbolic material figures of merit
Source: Sci Rep. 2024 Oct 24;14:25156. doi: 10.1038/s41598-024-74398-1 (PMC11502663; doi:10.1038/s41598-024-74398-1)
Supplement: Supplementary file 1 — Supplementary Material 1 [file 41598_2024_74398_MOESM1_ESM.pdf]

Supplementary Material: The role of losses in determining hyperbolic material figures of merit

E.M. Jackson[*,a], J.G. Tischler[b], D.C. Ratchford[a], C.T. Ellis[a]

[a]Naval Research Laboratory, Washington DC 20375

[b] Physics and Astronomy Department, University of Oklahoma, Norman, Oklahoma 73019

[b] Center for Quantum Research and Technology, University of Oklahoma, Norman, Oklahoma 73019

Derivation of Eq.2: Dispersion as function of propagation angle:

For simplicity, we restrict ourselves to uniaxial materials such that the permeability and permittivities are both diagonal with the same extraordinary axis defined to be along the z-axis. Then the material has cylindrical symmetry and the permittivity has components $\varepsilon_x = \varepsilon_y \equiv \varepsilon_r$ and ε_z. Similarly, the permeability components are μ_r, μ_z. Then, for a plane wave, Maxwell's equations yield the dispersion relation:

TM mode: $$k_0^2 = \frac{k_r^2}{\mu_r \varepsilon_z} + \frac{k_z^2}{\mu_r \varepsilon_r} \qquad \text{(S.1a)}$$

TE mode: $$k_0^2 = \frac{k_r^2}{\mu_z \varepsilon_r} + \frac{k_z^2}{\mu_r \varepsilon_r} \qquad \text{(S.1b)}$$

where $k_0 = \omega/c$ is the free space wavevector and k_r and k_z are the wavevectors in the plane (ordinary axes) and along z (extraordinary axis), respectively. The electric field of the TM mode is in the plane of incidence defined by k and z, and the B field is perpendicular to it. Similarly, for the TE mode, the B field is in the plane of incidence and the E field is perpendicular to it. The dispersion relations of both modes are described by the same functional form with the roles of ε and μ reversed. We therefore proceed by solving for the dispersion of the TM mode. The dispersion of the TE mode can be obtained by simple substitution μ_i for ε_i and vice versa.

Rewriting Eq. S.1a to explicitly show the imaginary terms we obtain:

$$k_0^2 = \frac{|k_r|^2 e^{i2\phi_r}}{|\mu_r| e^{i\gamma_{\mu r}} |\varepsilon_z| e^{i\gamma_{\varepsilon z}}} + \frac{|k_z|^2 e^{i2\phi_z}}{|\mu_r| e^{i\gamma_{\mu r}} |\varepsilon_r| e^{i\gamma_{\varepsilon r}}} = \frac{|k_r|^2 e^{i(2\phi_r - \gamma_{\varepsilon z} - \gamma_{\mu r})}}{|\mu_r||\varepsilon_z|} + \frac{|k_z|^2 e^{i(2\phi_z - \gamma_{\varepsilon r} - \gamma_{\mu r})}}{|\mu_r||\varepsilon_r|} \qquad \text{(S.2)}$$

where $\phi_{r,z}$ are the complex phases of the wavevectors and $\gamma_{\varepsilon r, \varepsilon z,}$ and $\gamma_{\mu r,\ \mu z}$ are the arguments of the dielectric permittivities and permeabilities, respectively. For simplicity we assume $\mu > 0$. Then the signs of the permittivities are reflected in the corresponding argument γ so for the two hyperbolic cases $0 \le \gamma_{\varepsilon z}$, $\gamma_{\mu r} \le \pi/2$ ($\pi/2 \le \gamma_{\varepsilon z} \le \pi$) and $\pi/2 \le \gamma_{\varepsilon r} \le \pi$ ($0 \le \gamma_{\varepsilon r}$, $\gamma_{\mu r} \le \pi/2$) results in Type 2 (Type 1) hyperbolic behavior.

The real and imaginary parts of k must be collinear, i.e. $|k_z|\sin(\phi_z)/|k_r|\sin(\phi_r) = |k_z|\cos(\phi_z)/|k_r|\cos(\phi_r)$. If not, the fields will grow exponentially in the direction perpendicular to the propagation, resulting in a divergence. This condition is satisfied when $\phi_z = \phi_r = \phi$, which yields the following relations for the real and imaginary components of Eq.S2:

$$\left|\mu_r\right| k_0^2 = \frac{\left|k_r\right|^2}{\left|\varepsilon_z\right|}\cos(2\phi - \gamma_{\varepsilon z} - \gamma_{\mu r}) + \frac{\left|k_z\right|^2}{\left|\varepsilon_r\right|}\cos(2\phi - \gamma_{\varepsilon r} - \gamma_{\mu r}) \qquad \text{(S.3a)}$$

$$0 = \frac{\left|k_r\right|^2}{\left|\varepsilon_z\right|}\sin(2\phi - \gamma_{\varepsilon z} - \gamma_{\mu r}) + \frac{\left|k_z\right|^2}{\left|\varepsilon_r\right|}\sin(2\phi - \gamma_{\varepsilon r} - \gamma_{\mu r}) \qquad \text{(S.3b)}$$

Equations 2 in the paper are derived from the equations above as follows:

First we expand the sin and cos terms in Eq. S.3b to obtain:

$$0 = \left[\varepsilon_r k_r^2 \cos(\gamma_{\varepsilon z} + \gamma_{\mu r}) + \varepsilon_z k_z^2 \cos(\gamma_{\varepsilon r} + \gamma_{\mu r})\right]\sin(2\phi) - \left[\varepsilon_r k_r^2 \sin(\gamma_{\varepsilon z} + \gamma_{\mu r}) + \varepsilon_z k_z^2 \sin(\gamma_{\varepsilon r} + \gamma_{\mu r})\right]\cos(2\phi)$$

Substituting $k_z = \left|k\right|\cos(\theta)$ and $k_r = \left|k\right|\sin(\theta)$ where θ is the standard polar angle gives:

$$0 = a\sin(2\phi) - b\cos(2\phi)$$

where it is convenient to define:

$$a \equiv \left|\varepsilon_r\right|\cos(\gamma_{\varepsilon z} + \gamma_{\mu r})\sin^2\theta + \left|\varepsilon_z\right|\cos(\gamma_{\varepsilon r} + \gamma_{\mu r})\cos^2\theta$$

$$b \equiv \left|\varepsilon_r\right|\sin(\gamma_{\varepsilon z} + \gamma_{\mu r})\sin^2\theta + \left|\varepsilon_z\right|\sin(\gamma_{\varepsilon r} + \gamma_{\mu r})\cos^2\theta$$

This immediately yields:

$$\tan(2\phi) = \frac{b}{a} = \frac{\varepsilon_r \sin(\gamma_{\varepsilon z} + \gamma_{\mu r})\sin^2(\theta) + \varepsilon_z \sin(\gamma_{\varepsilon r} + \gamma_{\mu r})\cos^2(\theta)}{\varepsilon_r \cos(\gamma_{\varepsilon z} + \gamma_{\mu r})\sin^2(\theta) + \varepsilon_z \cos(\gamma_{\varepsilon r} + \gamma_{\mu r})\cos^2(\theta)}. \qquad \text{(S.4a)}$$

Which is the same as Eq.2b of the main text. Similarly Eq. S.3a becomes:

$$\left|\mu_r\right|\left|\varepsilon_z\right|\left|\varepsilon_r\right| k_0^2 = \left|k\right|^2\left[a\cos(2\phi) + b\sin(2\phi)\right]$$

Writing this in terms of tan(2ϕ) allows the phase to be eliminated:

$$\begin{aligned}\left|\mu_r\right|\left|\varepsilon_z\right|\left|\varepsilon_r\right| k_0^2 &= \cos(2\phi)\left[a + b\tan(2\phi)\right]\left|k\right|^2 \\ &= \frac{1}{\sqrt{1+\tan^2(2\phi)}}\left[a + b\tan(2\phi)\right]\left|k\right|^2\end{aligned}$$

substituting from Eq. 4b we find:

$$\begin{aligned}\left|\mu_r\right|\left|\varepsilon_z\right|\left|\varepsilon_r\right| k_0^2 &= \frac{1}{\sqrt{1+\left(\frac{b}{a}\right)^2}}\left[a + \frac{b^2}{a}\right]\left|k\right|^2 \\ &= \left|k\right|^2\sqrt{a^2 + b^2}\end{aligned}$$

Inserting the definitions of *a* and *b* and solving for *k* we obtain.

$$\frac{\left|k\right|}{k_0} = \sqrt{\mu_r}\, f(\theta) = \sqrt{\mu_r}\left(\frac{\sin^4(\theta)}{\left|\varepsilon_z\right|^2} + \frac{\cos^4(\theta)}{\left|\varepsilon_r\right|^2} + 2\cos(\gamma_{\varepsilon z} - \gamma_{\varepsilon r})\frac{\cos^2(\theta)\sin^2(\theta)}{\left|\varepsilon_z\right|\left|\varepsilon_r\right|}\right)^{-1/4} \qquad \text{(S.4b)}$$

which is Eq. 2c of the main text.

Figs. S1 and S2 show examples using values of permittivity in Table I in the main text.

Figure S1

(a)

k_0 (1/cm)	ε_{r1}	ε_{r2}	ε_{z1}	ε_{z2}	μ_r, μ_z
871	3.25	0.0124	-21.57	68.35	1

(b)

k_0 (1/cm)	ε_{r1}	ε_{r2}	ε_{z1}	ε_{z2}	μ_r, μ_z
879	3.27	0.012	-2.226	0.226	1

(c)

k_0 (1/cm)	ε_{r1}	ε_{r2}	ε_{z1}	ε_{z2}	μ_r, μ_z
888	3.30	0.0124	-0.036	0.052	1

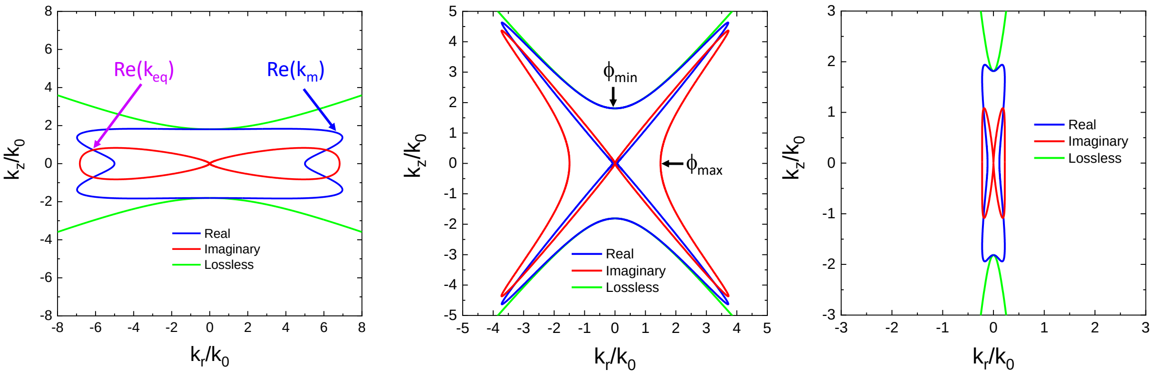

Fig. S1. Dispersion relations for calcite at selected frequencies in the Type I band.

Figure S2

(a)

k_0 (1/cm)	ε_{r1}	ε_{r2}	ε_{z1}	ε_{z2}	$\mu_r=\mu_z$
1405	-35.2	66.78	2.017	6.04E-04	1

(b)

k_0 (1/cm)	ε_{r1}	ε_{r2}	ε_{z1}	ε_{z2}	$\mu_r=\mu_z$
1500	-1.43	0.195	2.036	4.88E-04	1

(c)

k_0 (1/cm)	ε_{r1}	ε_{r2}	ε_{z1}	ε_{z2}	$\mu_r=\mu_z$
1550	-0.027	0.086	2.0444	4.39E-04	1

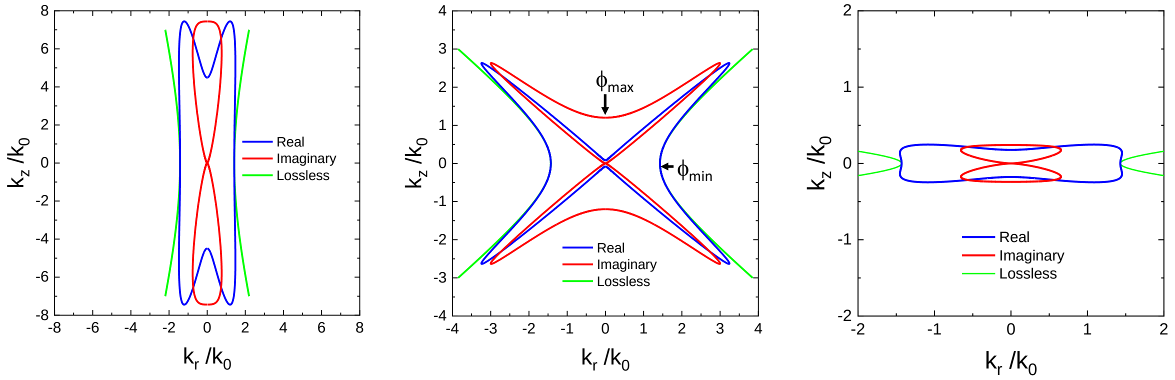

Fig. S2. Dispersion relations for calcite at selected frequencies in the Type II band.

Proof that $\phi(\theta)$ is monotonic over $0^\circ<\theta<90^\circ$:

We will prove that $\partial\phi/\partial\theta\neq 0$ over this range.

As before we define:

$$a \equiv |\varepsilon_r|\cos(\gamma_{\varepsilon z}+\gamma_{\mu r})\sin^2\theta + |\varepsilon_z|\cos(\gamma_{\varepsilon r}+\gamma_{\mu r})\cos^2\theta$$

$$b \equiv |\varepsilon_r|\sin(\gamma_{\varepsilon z}+\gamma_{\mu r})\sin^2\theta + |\varepsilon_z|\sin(\gamma_{\varepsilon r}+\gamma_{\mu r})\cos^2\theta$$

then

$$\frac{\partial a}{\partial\theta} = \left(|\varepsilon_r|\cos(\gamma_{\varepsilon z}+\gamma_{\mu r}) - |\varepsilon_z|\cos(\gamma_{\varepsilon r}+\gamma_{\mu r})\right)\sin\theta\cos\theta$$

$$\frac{\partial b}{\partial\theta} = \left(|\varepsilon_r|\sin(\gamma_{\varepsilon z}+\gamma_{\mu r}) - |\varepsilon_z|\sin(\gamma_{\varepsilon r}+\gamma_{\mu r})\right)\sin\theta\cos\theta$$

With these definitions we have already shown:

$$\tan(2\phi) = \frac{b}{a}.$$

then:

$$\frac{\partial\tan 2\phi}{\partial\theta} = \frac{2}{\cos^2 2\phi}\frac{\partial\phi}{\partial\theta} = \frac{1}{a}\frac{\partial b}{\partial\theta} - \frac{b}{a^2}\frac{\partial a}{\partial\theta}$$

$$2\frac{\partial\phi}{\partial\theta} = \frac{\cos^2 2\phi}{a^2}\left(a\frac{\partial b}{\partial\theta} - b\frac{\partial a}{\partial\theta}\right)$$

$$2\frac{\partial\phi}{\partial\theta} = \frac{1}{a^2}\frac{1}{1+(b/a)^2}\left(a\frac{\partial b}{\partial\theta} - b\frac{\partial a}{\partial\theta}\right)$$

simplifying and substituting for the derivatives we have:

$$2\frac{\partial\phi}{\partial\theta} = \frac{\sin\theta\cos\theta}{a^2+b^2}\Big[\left(|\varepsilon_r|\cos(\gamma_{\varepsilon z}+\gamma_{\mu r})\sin^2\theta + |\varepsilon_z|\cos(\gamma_{\varepsilon r}+\gamma_{\mu r})\cos^2\theta\right)\left(|\varepsilon_r|\sin(\gamma_{\varepsilon z}+\gamma_{\mu r}) - |\varepsilon_z|\sin(\gamma_{\varepsilon r}+\gamma_{\mu r})\right)$$
$$-\left(|\varepsilon_r|\sin(\gamma_{\varepsilon z}+\gamma_{\mu r})\sin^2\theta + |\varepsilon_z|\sin(\gamma_{\varepsilon r}+\gamma_{\mu r})\cos^2\theta\right)\left(|\varepsilon_r|\cos(\gamma_{\varepsilon z}+\gamma_{\mu r}) - |\varepsilon_z|\cos(\gamma_{\varepsilon r}+\gamma_{\mu r})\right)\Big]$$

$$2\frac{\partial\phi}{\partial\theta} = \frac{\sin\theta\cos\theta}{a^2+b^2}\Big[\left(|\varepsilon_z||\varepsilon_r|\sin(\gamma_{\varepsilon z}+\gamma_{\mu r})\cos(\gamma_{\varepsilon r}+\gamma_{\mu r})\cos^2\theta - |\varepsilon_z||\varepsilon_r|\cos(\gamma_{\varepsilon z}+\gamma_{\mu r})\sin(\gamma_{\varepsilon r}+\gamma_{\mu r})\sin^2\theta\right)$$
$$-\left(|\varepsilon_z||\varepsilon_r|\cos(\gamma_{\varepsilon z}+\gamma_{\mu r})\sin(\gamma_{\varepsilon r}+\gamma_{\mu r})\cos^2\theta - |\varepsilon_r||\varepsilon_z|\sin(\gamma_{\varepsilon z}+\gamma_{\mu r})\cos(\gamma_{\varepsilon r}+\gamma_{\mu r})\sin^2\theta\right)\Big]$$

$$2\frac{\partial\phi}{\partial\theta} = \frac{\sin\theta\cos\theta|\varepsilon_z||\varepsilon_r|}{a^2+b^2}\left(\sin(\gamma_{\varepsilon z}+\gamma_{\mu r})\cos(\gamma_{\varepsilon r}+\gamma_{\mu r}) - \cos(\gamma_{\varepsilon z}+\gamma_{\mu r})\sin(\gamma_{\varepsilon r}+\gamma_{\mu r})\right)$$

$$2\frac{\partial\phi}{\partial\theta} = \frac{\sin\theta\cos\theta|\varepsilon_z||\varepsilon_r|}{a^2+b^2}\sin(\gamma_{\varepsilon z}-\gamma_{\varepsilon r})$$

We have already shown that :

$$|\mu_r||\varepsilon_z||\varepsilon_r|k_0^2 = |k|^2\sqrt{a^2+b^2}$$

then since |k|>0 we also have $\left(a^2+b^2\right)>0$

therefore $\frac{\partial\phi}{\partial\theta}=0$ only if $\sin(\gamma_{\varepsilon z}-\gamma_{\varepsilon r})=0$ or sinθcosθ=0, i.e. when θ=0° and θ=90°.

$\sin(\gamma_{\varepsilon z}-\gamma_{\varepsilon r})=0$ is only possible in elliptical materials. In this case γ_ε is constant and the solution of Eq. S1a is trivial. Otherwise, from Eq.S.4a we see that ϕ(θ)=ϕ(-θ) and ϕ(90+δθ)=ϕ(90-δθ). Therefore θ=0° and θ=90° are always local extrema.

Furthermore, the sign of $\frac{\partial\phi}{\partial\theta}$ is determined by the sign of $\sin(\gamma_{\varepsilon z}-\gamma_{\varepsilon r})$

Type 1: $\sin\left(\gamma_{\varepsilon z}-\gamma_{\varepsilon r}\right)>0$, ϕ has a minimum at θ=0° and a maximum at 90°

Type 2: $\sin\left(\gamma_{\varepsilon z}-\gamma_{\varepsilon r}\right)<0$, ϕ has a minimum at θ=90° and a maximum at 0°

Derivation of Eq.3: Dispersion as function of phase:

From the previous derivation we have the results:

$$|\mu_r||\varepsilon_z||\varepsilon_r|k_0^2 = |k|^2\sqrt{a^2+b^2}$$

$$1 = \left(\frac{|k|^2}{|\mu_r||\varepsilon_z||\varepsilon_r|k_0^2}\right)^2\left(a^2+b^2\right)$$

and

$$\tan(2\phi) = \frac{b}{a}$$

We identify:

$$\sin(2\phi) = \frac{|k|^2}{|\mu_r||\varepsilon_z||\varepsilon_r|k_0^2}b = \frac{|k|^2}{|\mu_r||\varepsilon_z||\varepsilon_r|k_0^2}\left(|\varepsilon_r|\sin(\gamma_{\varepsilon z}+\gamma_{\mu r})\sin^2\theta + |\varepsilon_z|\sin(\gamma_{\varepsilon r}+\gamma_{\mu r})\cos^2\theta\right)$$

$$\cos(2\phi) = \frac{|k|^2}{|\mu_r||\varepsilon_z||\varepsilon_r|k_0^2}a = \frac{|k|^2}{|\mu_r||\varepsilon_z||\varepsilon_r|k_0^2}\left(|\varepsilon_r|\cos(\gamma_{\varepsilon z}+\gamma_{\mu r})\sin^2\theta + |\varepsilon_{\varepsilon z}|\cos(\gamma_{\varepsilon r}+\gamma_{\mu r})\cos^2\theta\right)$$

We replace the $\cos^2(\theta)$ by 1-$\sin^2(\theta)$ in the $\sin(2\phi)$ and $\cos(2\phi)$ equations and isolate the $\sin^2(\theta)$ term to obtain:

$$\frac{1}{|\varepsilon_r|\cos(\gamma_{\varepsilon z}+\gamma_{\mu r}) - |\varepsilon_z|\cos(\gamma_{\varepsilon r}+\gamma_{\mu r})}\cos(2\phi) = \frac{|k|^2}{|\mu_r||\varepsilon_r||\varepsilon_z|k_0{}^2}\left[\frac{\cos(\gamma_{\varepsilon r}+\gamma_{\mu r})|\varepsilon_z|}{|\varepsilon_r|\cos(\gamma_{\varepsilon z}+\gamma_{\mu r}) - |\varepsilon_z|\cos(\gamma_{\varepsilon r}+\gamma_{\mu r})} + \sin^2(\theta)\right]$$

$$\frac{1}{|\varepsilon_r|\sin(\gamma_{\varepsilon z}+\gamma_{\mu r}) - |\varepsilon_z|\sin(\gamma_{\varepsilon r}+\gamma_{\mu r})}\sin(2\phi) = \frac{|k|^2}{|\mu_r||\varepsilon_r||\varepsilon_z|k_0{}^2}\left[\frac{\sin(\gamma_{\varepsilon r}+\gamma_{\mu r})|\varepsilon_z|}{|\varepsilon_r|\sin(\gamma_{\varepsilon z}+\gamma_{\mu r}) - |\varepsilon_z|\sin(\gamma_{\varepsilon r}+\gamma_{\mu r})} + \sin^2(\theta)\right]$$

We can now eliminate $\sin(\theta)$. After some algebra we obtain the magnitude of k:

$$\left|\frac{k}{k_0}\right| = \sqrt{|\mu_r|}\sqrt{\frac{\left(|\varepsilon_r|\sin(\gamma_{\varepsilon z}+\gamma_{\mu r}) - |\varepsilon_z|\sin(\gamma_{\varepsilon r}+\gamma_{\mu r})\right)}{\sin(\gamma_{\varepsilon z}-\gamma_{\varepsilon r})}\cos(2\phi) - \frac{\left(|\varepsilon_r|\cos(\gamma_{\varepsilon z}+\gamma_{\mu r}) - |\varepsilon_z|\cos(\gamma_{\varepsilon r}+\gamma_{\mu r})\right)}{\sin(\gamma_{\varepsilon z}-\gamma_{\varepsilon r})}\sin(2\phi)}$$

Then $k/k_0 = |k/k_0|\,e^{i\phi}$

$$\frac{k}{k_0} = \sqrt{|\mu_r|}e^{i\phi}\sqrt{\frac{\left(|\varepsilon_r|\sin(\gamma_{\varepsilon z}+\gamma_{\mu r}) - |\varepsilon_z|\sin(\gamma_{\varepsilon r}+\gamma_{\mu r})\right)}{\sin(\gamma_{\varepsilon z}-\gamma_{\varepsilon r})}\cos(2\phi) - \frac{\left(|\varepsilon_r|\cos(\gamma_{\varepsilon z}+\gamma_{\mu r}) - |\varepsilon_z|\cos(\gamma_{\varepsilon r}+\gamma_{\mu r})\right)}{\sin(\gamma_{\varepsilon z}-\gamma_{\varepsilon r})}\sin(2\phi)} \qquad \text{(S.5)}$$

which is Eq.3a of the main text.

From Eq. S.4a we find that the phase ϕ equals $(\gamma_{\varepsilon r}+\gamma_{\mu r})/2$ at $\theta=0^o$ and $(\gamma_{\varepsilon z}+\gamma_{\mu r})/2$ at $\theta=90^o$. It increases (Type I) or decreases (Type II) monotonically with θ between these 2 points. Therefore Eq. S.5 reproduces the 1st quadrant of the dispersion curve for Type I materials with $\phi_{min}\equiv(\gamma_{\varepsilon r}+\gamma_{\mu r})/2\leq\phi\leq(\gamma_{\varepsilon z}+\gamma_{\mu r})/2\equiv\phi_{max}$. ϕ_{min} and ϕ_{max} are reversed for Type 2 materials, i.e. $\phi_{min}\equiv(\gamma_{\varepsilon z}+\gamma_{\mu r})/2$ and $\phi_{max}\equiv(\gamma_{\varepsilon r}+\gamma_{\mu r})/2$.

Derivation of Eq.4: the phase at the maximum of Re(k):

The maximum of Re(k) is found in the usual way by setting the derivative to zero.

From Eq.S.5 we have:

$$\frac{k}{k_0} = \sqrt{|\mu_r|}e^{i\phi}\sqrt{\frac{\left(|\varepsilon_r|\sin(\gamma_{\varepsilon z}+\gamma_{\mu r})-|\varepsilon_z|\sin(\gamma_{\varepsilon r}+\gamma_{\mu r})\right)}{\sin(\gamma_{\varepsilon z}-\gamma_{\varepsilon r})}\cos(2\phi)-\frac{\left(|\varepsilon_r|\cos(\gamma_{\varepsilon z}+\gamma_{\mu r})-|\varepsilon_z|\cos(\gamma_{\varepsilon r}+\gamma_{\mu r})\right)}{\sin(\gamma_{\varepsilon z}-\gamma_{\varepsilon r})}\sin(2\phi)}$$

we define:

$$\mathrm{u}=\left(|\varepsilon_r|\sin(\gamma_{\varepsilon z}+\gamma_{\mu r})-|\varepsilon_z|\sin(\gamma_{\varepsilon r}+\gamma_{\mu r})\right)$$

$$v=\left(|\varepsilon_r|\cos(\gamma_{\varepsilon z}+\gamma_{\mu r})-|\varepsilon_z|\cos(\gamma_{\varepsilon r}+\gamma_{\mu r})\right)$$

Then:

$$\frac{k}{k_0} = \sqrt{\frac{|\mu_r|}{\sin(\gamma_{\varepsilon z}-\gamma_{\varepsilon r})}}e^{i\phi}\sqrt{u\cos(2\phi)-v\sin(2\phi)}$$

We set the derivative of the real part of k=kcos(ϕ) with respect to ϕ to 0:

$$\left.\frac{\partial\left(k\cos\phi\right)}{\partial\phi}\right|_{\phi_{km}} = 0 = \frac{1}{2\sqrt{\sin(\gamma_{\varepsilon z}-\gamma_{\varepsilon r})}}\frac{u\left[-4\cos^3(\phi)\sin(\phi)+2\cos(\phi)\sin^3(\phi)-2\cos^3(\phi)\sin(\phi)\right]-2v\left[\cos^4(\phi)-3\cos^2(\phi)\sin^2(\phi)\right]}{\sqrt{u\left(\cos^4(\phi)-\cos^2(\phi)\sin^2(\phi)\right)-v2\cos^3(\phi)\sin(\phi)}}$$

This simplifies to:

$$0 = 4\sin^3(\phi_{\mathrm{km}})-3\sin(\phi_{km})\pm\frac{v}{\sqrt{v^2+u^2}}$$

There are 6 solutions, but the correct solution in the 1st quadrant is:

$$\sin(\phi_{km}) = \cos\left(\frac{2\pi+\arctan\left(\frac{u}{v}\right)}{3}\right)$$

$$\phi_{km} = \frac{\pi}{6}+\frac{\arctan\left(\frac{u}{v}\right)}{3}$$

$$\phi_{km} = \frac{\pi}{6}+\frac{\arctan\left(\Delta\right)}{3} \qquad \text{(S.6a)}$$

where

$$\Delta=\frac{u}{v}=\frac{|\varepsilon_r|\sin(\gamma_{\varepsilon z}+\gamma_{\mu r})-|\varepsilon_z|\sin(\gamma_{\varepsilon r}+\gamma_{\mu r})}{|\varepsilon_r|\cos(\gamma_{\varepsilon z}+\gamma_{\mu r})-|\varepsilon_z|\cos(\gamma_{\varepsilon r}+\gamma_{\mu r})} \qquad \text{(S.6b)}$$

which is Eq.4b of the main text.

If ϕ_{km} is not between $(\gamma_{\varepsilon r}+\gamma_{\mu r})/2$ and $(\gamma_{\varepsilon z}+\gamma_{\mu r})/2$ then the maximum of Re(k) is one of the end points, i.e. $\theta=0^o$ or $\theta=90^o$. This is usually the case in the ellipsoidal regime, but rarely true in the hyperbolic regimes.

Derivation of Eq.5: the maximum of Re(k):

Substituting ϕ_{km} into Eq. S.5 gives:

From Eq. S.5:

$$\frac{k}{k_0}=\sqrt{|\mu_r|}e^{i\phi}\sqrt{\frac{\left(|\varepsilon_r|\sin(\gamma_{\varepsilon z}+\gamma_{\mu r})-|\varepsilon_z|\sin(\gamma_{\varepsilon r}+\gamma_{\mu r})\right)}{\sin(\gamma_{\varepsilon z}-\gamma_{\varepsilon r})}\cos(2\phi)-\frac{\left(|\varepsilon_r|\cos(\gamma_{\varepsilon z}+\gamma_{\mu r})-|\varepsilon_z|\cos(\gamma_{\varepsilon r}+\gamma_{\mu r})\right)}{\sin(\gamma_{\varepsilon z}-\gamma_{\varepsilon r})}\sin(2\phi)}$$

Taking the real part of k and substituting in ϕ_{km}:

$$\mathrm{Max}\left(\mathrm{Re}\left(\frac{k}{k_0}\right)\right)=\sqrt{|\mu_r|}\cos\phi_{\mathrm{km}}\sqrt{\frac{\left(|\varepsilon_r|\sin(\gamma_{\varepsilon z}+\gamma_{\mu r})-|\varepsilon_z|\sin(\gamma_{\varepsilon r}+\gamma_{\mu r})\right)}{\sin(\gamma_{\varepsilon z}-\gamma_{\varepsilon r})}\cos(2\phi_{\mathrm{km}})+\frac{\left(|\varepsilon_z|\cos(\gamma_{\varepsilon r}+\gamma_{\mu r})-|\varepsilon_r|\cos(\gamma_{\varepsilon z}+\gamma_{\mu r})\right)}{\sin(\gamma_{\varepsilon z}-\gamma_{\varepsilon r})}\sin(2\phi_{\mathrm{km}})}$$

$$\mathrm{Max}\left(\mathrm{Re}\left(\frac{k}{k_0}\right)\right)=\sqrt{|\mu_r|}\cos\phi_{\mathrm{km}}\sqrt{\left(|\varepsilon_z|\cos(\gamma_{\varepsilon r}+\gamma_{\mu r})-|\varepsilon_r|\cos(\gamma_{\varepsilon z}+\gamma_{\mu r})\right)\left(\frac{\Delta\cos(2\phi_{\mathrm{km}})+\sin(2\phi_{\mathrm{km}})}{\sin(\gamma_{\varepsilon z}-\gamma_{\varepsilon r})}\right)}$$

For small Δ:

$$\mathrm{Max}\left(\mathrm{Re}\left(\frac{k}{k_0}\right)\right)\approx\sqrt{|\mu_r|}\cos\phi_{\mathrm{km}}\sqrt{\frac{\left(|\varepsilon_z|\cos(\gamma_{\varepsilon r}+\gamma_{\mu r})-|\varepsilon_r|\cos(\gamma_{\varepsilon z}+\gamma_{\mu r})\right)}{\sin(\gamma_{\varepsilon z}-\gamma_{\varepsilon r})}\sin(2\phi_{\mathrm{km}})}$$

for small Δ, $\phi_{\mathrm{km}}\approx\pi/6$ so $\cos(\phi_{\mathrm{km}})\approx\sin(2\phi_{\mathrm{km}})\approx\sqrt{3}/2$ and we obtain:

$$\mathrm{Max}\left(\mathrm{Re}\left(\frac{k}{k_0}\right)\right)\approx\sqrt{|\mu_r|}\frac{3^{3/4}}{\sqrt{2}}\sqrt{\frac{\left(|\varepsilon_z|\cos(\gamma_{\varepsilon r}+\gamma_{\mu r})-|\varepsilon_r|\cos(\gamma_{\varepsilon z}+\gamma_{\mu r})\right)}{\sin(\gamma_{\varepsilon z}-\gamma_{\varepsilon r})}} \qquad \text{(S.7)}$$

which is Eq.5a of the main text.

In hyperbolic materials the cosine terms always have opposite signs. Then to 1st order the cosines become ±1. The denominator can be written:

$$\sin(\gamma_{\varepsilon z}-\gamma_{\varepsilon r})=\sin(\gamma_{\varepsilon z})\cos(\gamma_{\varepsilon r})-\cos(\gamma_{\varepsilon z})\sin(\gamma_{\varepsilon r})$$

Again to 1st order the sines are $\sin(\gamma_i)\approx|\varepsilon_{i2}/\varepsilon_{i1}|$. Then we obtain Eq. S.8:

$$\mathrm{Max}\left(\mathrm{Re}\left(\frac{k}{k_0}\right)\right)\equiv\mathrm{Re}\left(\frac{k_m}{k_0}\right)\approx\frac{3^{3/4}\sqrt{\mu_r}}{2\sqrt{2}}\sqrt{\frac{|\varepsilon_z|+|\varepsilon_r|}{|\varepsilon_{r2}/\varepsilon_{r1}|+|\varepsilon_{z2}/\varepsilon_{z1}|}} \qquad \text{(S.8)}$$

which is Eq.5b of the main text.

Derivation of Eqs. 6& 7: Scaling of L_p vs. λ_{eq}:

We first compute k(ϕ) at ϕ=π/4 from Eq.S5:

$$\frac{k}{k_0}=\sqrt{|\mu_r|}e^{i\phi}\sqrt{\frac{\left(|\varepsilon_r|\sin(\gamma_{\varepsilon z}+\gamma_{\mu r})-|\varepsilon_z|\sin(\gamma_{\varepsilon r}+\gamma_{\mu r})\right)}{\sin(\gamma_{\varepsilon z}-\gamma_{\varepsilon r})}\cos(2\phi)-\frac{\left(|\varepsilon_r|\cos(\gamma_{\varepsilon z}+\gamma_{\mu r})-|\varepsilon_z|\cos(\gamma_{\varepsilon r}+\gamma_{\mu r})\right)}{\sin(\gamma_{\varepsilon z}-\gamma_{\varepsilon r})}\sin(2\phi)}$$

At ϕ=π/4, cos(2ϕ)=0 and sin(2ϕ)=1 so:

$$\frac{k_{eq}}{k_0}\equiv\frac{k\left(\frac{\pi}{4}\right)}{k_0}=\sqrt{|\mu_r|}e^{i\frac{\pi}{4}}\sqrt{-\frac{\left(|\varepsilon_r|\cos(\gamma_{\varepsilon z}+\gamma_{\mu r})-|\varepsilon_z|\cos(\gamma_{\varepsilon r}+\gamma_{\mu r})\right)}{\sin(\gamma_{\varepsilon z}-\gamma_{\varepsilon r})}}$$

Then Re(k_{eq})/Re(k) is:

$$\frac{\lambda}{\lambda_{eq}}=\frac{\mathrm{Re}(k_{eq})}{\mathrm{Re}(k)}=\frac{\cos(\pi/4)}{\cos(\phi)}\sqrt{\frac{-\left(\varepsilon_r\cos(\gamma_{\varepsilon z}+\gamma_{\mu r})-\varepsilon_z\cos(\gamma_{\varepsilon r}+\gamma_{\mu r})\right)}{\left(\varepsilon_r\sin(\gamma_{\varepsilon z}+\gamma_{\mu r})-\varepsilon_z\sin(\gamma_{\varepsilon r}+\gamma_{\mu r})\right)\cos(2\phi)-\left(\varepsilon_r\cos(\gamma_{\varepsilon z}+\gamma_{\mu r})-\varepsilon_z\cos(\gamma_{\varepsilon r}+\gamma_{\mu r})\right)\sin(2\phi)}}$$

Dividing the numerator and denominator of the radical by $\left(\varepsilon_r\cos(\gamma_{\varepsilon z}+\gamma_{\mu r})-\varepsilon_z\cos(\gamma_{\varepsilon r}+\gamma_{\mu r})\right)$ and using the definition of $\Delta=\dfrac{\varepsilon_r\sin(\gamma_{\varepsilon z}+\gamma_{\mu r})-\varepsilon_z\sin(\gamma_{\varepsilon r}+\gamma_{\mu r})}{\varepsilon_r\cos(\gamma_{\varepsilon z}+\gamma_{\mu r})-\varepsilon_z\cos(\gamma_{\varepsilon r}+\gamma_{\mu r})}$ we find:

$$\frac{\lambda}{\lambda_{eq}}=\frac{\mathrm{Re}(k_{eq})}{\mathrm{Re}(k)}=\frac{1}{\sqrt{2}\cos(\phi)}\sqrt{\frac{-1}{\Delta\cos(2\phi)-\sin(2\phi)}}$$

$$\frac{\lambda}{\lambda_{eq}}=\frac{1}{\sqrt{2}}\frac{1}{\cos(\phi)\sqrt{\sin(2\phi)-\Delta\cos(2\phi)}} \qquad \text{(S.9a)}$$

which is Eq. 7a of the main text

Since Re(k)=cot(ϕ)Im(k) we also have:

$$\frac{\mathrm{Re}(k_{eq})}{\mathrm{Im}(k)}=\frac{2\pi L_p}{\lambda_{\min}}=\cot(\phi)\frac{\lambda}{\lambda_{eq}} \quad \text{so:}$$

$$\frac{2\pi L_p}{\lambda_{eq}}=\frac{1}{\sqrt{2}}\frac{1}{\sin(\phi)\sqrt{\sin(2\phi)-\Delta\cos(2\phi)}} \qquad \text{(S.9b)}$$

which is Eq. 7b of the main text.
